# Notes and Notices

**Published:** 1896-06

**Authors:** 


### NOTES AND NOTICES.

Dr. L. D. Rogers and wife, of Chicago, have gone to Europe for the summer. They sailed May 29th.

Dr. B. L. B. Baylies announces that he has removed his down-town office from 174 Schermerhorn Street to 133 Remsen, near Clinton Street, Brooklyn. Telephone, 398 Bedford.

The Manhattan Life Insurance Company, of New York (No. 66 Broadway) has recognized the homœopathic school of medicine by appointing upon its staff of medical examiners Drs. Eugene H. Porter and St. Clair Smith.

"Keep Your Eyes Open."—"Having eyes, see not." That's the trouble with lots of people, who never see the wonders of their own dooryard or farm, the roadside by which they drive, the fields and woods through which they walk, or the ponds where they catch anything from frogs to black bass. Of many of the usually unseen "wonders and beauties of Nature" you can read in *The Observer*, Portland, Conn. This very interesting illustrated magazine is of interest to all students and lovers of Nature. It assists parents in providing home interests for their children, and leads the "children of a larger growth," to be more interested in home. Do you enjoy roaming over hills and fields or through the woods; are you interested in birds, flowers, insects, rocks, etc.? then send ten cents for sample copy of *The Observer* (an illustrated monthly magazine), Portland, Conn. Subscription, $1.00 a year. Give it a fair trial as to its merits.

The Pennsylvania State College.—Special attention is invited to the following points: 1. The fall session of 1896–7 opens September 16th, 1896 (not September 9th). 2. Examinations for admission will be held at the College June 18th and September 15th, 1896. 3. Local examinations will be held Wednesday, June 24th, as follows: At Philadelphia, Reading, Harrisburg, and Williamsport, at the rooms of the Y. M. C. A.; at Scranton, at the School of the Lackawanna; at Pittsburg, at the rooms of the Central Board of Education—all beginning at 9 o'clock a. m. 4. Department for Women. With the establishment of a full classical course and elective courses in language, literature, history, and philosophy, the College now offers special advantages to young women, who are admitted to all classes on the same terms as young men. A separate cottage has been erected for their use, which furnishes an attractive and beautiful home. For catalogues or other information, address the President, State College, Centre County, Pa.

The Maywood Bicycle.—Chicago, Ill., Feb. 11th, 1896. I am still riding the "Maywood" Bicycle bought of you season before last, and shall ride it again this year. During the past two seasons I have covered on this wheel 13,256 miles, without a break, and total cost of repairs for spokes 45 cents.

Last September I rode from Chicago to Detroit, Mich., thence to Cincinnati, Ohio; thence to Atlanta, Ga., and the "Maywood" carried me through without any trouble. I never lose the opportunity to speak in praise of your wheel, for I believe there is none better. Will C. Davis, 358–366 Dearborn Street.

Pneumatic Truss Pads.—Those who are obliged to wear trusses have suffered from pads that are supposed to hold up the ruptured parts, and to alleviate the pain thus caused, hard and soft pads have been devised, and all proven more or less unsatisfactory.

A pneumatic truss pad that is non-collapsible has been invented by G. W. Flavell, and can be used on any truss. It has been found to correct all the difficulties of the old pads and gives instant relief.

One of the new pads should be in every physician's office, and a sample can be obtained at the nominal price of 50 cents, from G. W. Flavell & Bro., 1005 Spring Garden Street, Philadelphia, Pa.

Phenol Dressings.—The medical expert of the *Herald's* European edition reports several mishaps from the imprudent use of phenol dressing which were recently brought to the attention of the Paris Academie de Medecine. In one of the cases quoted in Paris a cook, after wounding her thumb in cleaning a pane of glass, and after keeping it dressed two days with a two per cent. solution of phenol, found that the second joint had become quite black; but, in spite of the acute pain, she continued to apply the solution. The caustic action was so rapid that four days from the beginning the second joint was black and shrunken, and subsequently dropped off. The *Herald's* foreign medical correspondent, commenting upon this result, says:

"The only means of protecting ourselves against accidents of this nature is to be well aware that strong carbolic solutions are not the only ones to be suspected; a phenol dressing one in forty, fifty, or even a hundred, kept around a finger or toe for one, two, or three days may cause more or less complete mortification of the whole or part of it, at any age and without any pathological predisposition, because the fact of being shut in by an impermeable wrapping gives to the dressing a degree of caustic power which the concentration of the solution would in no way lead us to expect."—*New York Herald.*

Advertising Axioms.—By J. Walter Thompson, of New York.—"The better the day, the better the deed." The better the "ad." and the better the mediums used, the better the results.

If you have something that the people need, advertise "with courage and faith," and the people at home and abroad will respond to your profit.

Do not forget that an advertisement in "perpetual motion," *if it is good*, will wear its way into the people's memory with consequent results to you.

Here is a suggestion—"*Make your advertisement an argument deriving its force from the situation, and present it clearly to all to whom it is addressed.*"

By advertisers I mean those who *know* that advertising *well done* is bound to bring results; by business men I mean a very large class of manufacturers who are "poor in the midst of great wealth"—*i. e., of possibilities of development.*

